# Contemporary experience with high-dose interleukin-2 therapy and impact on survival in patients with metastatic melanoma and metastatic renal cell carcinoma

**DOI:** 10.1007/s00262-016-1910-x

**Published:** 2016-10-06

**Authors:** Ajjai Alva, Gregory A. Daniels, Michael K. K. Wong, Howard L. Kaufman, Michael A. Morse, David F. McDermott, Joseph I. Clark, Sanjiv S. Agarwala, Gerald Miletello, Theodore F. Logan, Ralph J. Hauke, Brendan Curti, John M. Kirkwood, Rene Gonzalez, Asim Amin, Mayer Fishman, Neeraj Agarwal, James N. Lowder, Hong Hua, Sandra Aung, Janice P. Dutcher

**Affiliations:** 1 University of Michigan, Ann Arbor, MI USA; 2 Moores Cancer Center, University of California San Diego, La Jolla, CA USA; 3 University of Southern California, Los Angeles, CA USA; 4 Present Address: M.D. Anderson Cancer Center, Houston, TX USA; 5 Rutgers Cancer Center Institute of New Jersey, New Brunswick, NJ USA; 6 Duke University Medical Center, Durham, NC USA; 7 Beth Israel Deaconess Medical Center, Boston, MA USA; 8 Loyola University Medical Center, Maywood, IL USA; 9 St. Luke’s University Health Network and Temple University, Bethlehem, PA USA; 10 Hematology/Oncology Clinic, Baton Rouge, LA USA; 11 Indiana University Simon Cancer Center, Indianapolis, IN USA; 12 Nebraska Cancer Specialists, Omaha, NE USA; 13 Providence Portland Medical Center, Portland, OR USA; 14 Hillman Cancer Center Research, University of Pittsburgh Cancer Institute, Pavillion L1 32c, Pittsburgh, PA USA; 15 University of Colorado Cancer Center, Aurora, CO USA; 16 Levine Cancer Institute, Charlotte, NC USA; 17 Moffitt Cancer Center, Tampa, FL USA; 18 Huntsman Cancer Institute, University of Utah, Salt Lake City, UT USA; 19 Astex Pharmaceuticals, Pleasanton, CA USA; 20 Prometheus Laboratories Inc., San Diego, CA USA; 21 Present Address: Nektar Therapeutics, San Francisco, CA USA; 22 Cancer Research Foundation, Chappaqua, NY USA

**Keywords:** Immunotherapy, Melanoma, Renal cell carcinoma, Interleukin-2

## Abstract

High-dose interleukin-2 (HD IL-2) was approved for treatment of metastatic renal cell carcinoma (mRCC) in 1992 and for metastatic melanoma (mM) in 1998, in an era predating targeted therapies and immune checkpoint inhibitors. The PROCLAIM^SM^ registry was established to collect and analyze data for patients treated with HD IL-2 in the current era. This analysis includes 170 patients with mM and 192 patients with mRCC treated between 2005 and 2012 with survival data current as of July 27, 2015. For patients with mM, complete response (CR) was observed in 5 %, partial response (PR) in 10 %, stable disease (SD) in 22 %, and 63 % had progressive disease (PD). The median overall survival (mOS) for these patients was 19.6 months, with a median follow-up of 43.1 months. The mOS was not reached for patients achieving CR or PR, and was 33.4 months for patients with SD. For patients with mRCC, 6 % achieved CR, 9 % had PR, 22 % had SD, and 62 % had PD. The mOS was 41 months, with a median follow-up of 46.6 months. The mOS for patients who had CR and PR was not reached and was 49.6 months for patients with SD. There were no treatment-related deaths among 362 patients. The duration of mOS for patients with mM and mRCC is longer than historically reported. These data support a continued role for IL-2 in the treatment of eligible patients with mM or mRCC and warrant further evaluation of HD IL-2 in combination or sequence with other therapeutic agents.

**Electronic supplementary material:**

The online version of this article (doi:10.1007/s00262-016-1910-x) contains supplementary material, which is available to authorized users.

## Introduction

Historically, treatment options for patients with metastatic melanoma (mM) and metastatic renal cell carcinoma (mRCC) were limited and the prognosis was poor with 5-year overall survival (OS) of 5–10 %, respectively [1]. The finding that some patients with these malignancies responded to high-dose interleukin-2 (HD IL-2) ultimately led to Food and Drug Administration (FDA) approval of HD IL-2 for mRCC in 1992 and then for mM in 1998. The historical response rates and overall survival reported at the time of approval were 16 % and 11.4 months for mM and 15 % and 16.3 months for mRCC, respectively [2–4]. The trials that led to FDA approval were single arm, uncontrolled studies and early on were associated with significant therapy-related deaths, 2 and 4 %, respectively, in melanoma and renal cell carcinoma. They were not analyzed for the survival impact of stable disease.

Recent studies have reported improved survival for patients with both malignancies treated with HD IL-2, with reduced treatment-related mortality and a survival benefit associated with stable disease. A retrospective analysis performed at Providence Portland Cancer Center, which included 314 mM and 186 mRCC patients treated between 1997 and 2012, reported an objective response rate (ORR) of 28 % for mM and 24 % for mRCC patients [5]. In a study of 88 mRCC patients treated at Roswell Park between 2004 and 2011, the observed mOS was 35.5 months compared to historical reference range of 16–20 months [6]. In a prospective multicenter study conducted by the Cytokine Working Group, researchers reported an ORR of 25 % and mOS of 42.8 months in 120 mRCC patients treated between 2006 and 2009 [7]. Thus, the contemporary experience with HD IL-2 suggests improved overall response rate and survival in mM and mRCC compared to the historical data.

Following the initial observation of durable long-term benefit from HD IL-2, novel immunotherapies and targeted therapies (TT) have been developed for the treatment of both mM and mRCC. Immune checkpoint inhibitors directed against cytotoxic T lymphocyte antigen 4 (CTLA-4) such as ipilimumab [2011] [8, 9] or programmed cell death receptor-1 (PD-1) such as nivolumab [2014 for melanoma, 2015 for mRCC] and pembrolizumab [2014] [10–13] have demonstrated objective responses and durable remissions in mM and mRCC. In parallel with these advances in immunotherapy, the ability to target drivers of the mitogen-activated protein kinase (MAPK) pathway (vemurafenib [2011], dabrafenib [2013], trametinib [2013], cobimetinib [2015]) led to potential treatments for a genetically defined subset of the mM population [14–17]. For mRCC, inhibitors of the vascular endothelial growth factor (VEGF) pathway (axitinib [2012], sorafenib [2005], sunitinib [2006], bevacizumab [2009], pazopanib [2009] and cabozantinib [2016]) and inhibitors of the mammalian target of rapamycin (mTOR) pathway (temsirolimus [2007] and everolimus [2009]) have been approved [18–21]. Checkpoint inhibitors are effective as monotherapy in approximately 25 % of advanced melanoma patients, as yet undefined, and carry the risk of potentially serious toxicities [22, 23]. Molecular-targeted therapies such as vemurafenib show initial high response rates but patients quickly progress due to acquired drug resistance [24–26]. Although treatment options for mM and mRCC have increased in number, the optimal combination and sequencing of therapeutic modalities remains an area of intense investigation.

We hypothesized that real-world data describing the experience with HD IL-2 in the contemporary era of checkpoint inhibitors and targeted therapies would better define the relevance of HD IL-2 in the context of these new therapies. In an effort to study the real-world use of HD IL-2, an observational database, termed PROCLAIM^SM^ (PROLEUKIN^®^ Observational Study to Evaluate the Treatment Patterns and Clinical Response in Malignancy) was created in 2011 [27]. PROCLAIM is the largest patient registry in the world collecting data on IL-2 treatment outcomes, prospectively from about 40 enrolled sites. The PROCLAIM registry is designed to generate new hypotheses from retrospective and prospective cohort analyses. Herein, we report survival and outcomes data from the retrospective cohort analysis, from the 16 sites that provided retrospective data, as of July 27, 2015 (patients treated between 2005 and 2012).

## Materials and methods

### Patient cohort

Within the PROCLAIM registry (ClinicalTrials.gov identifier: NCT01415167) [27], established in 2011, retrospective data from de-identified patient cases have been abstracted from existing charts of patients treated with HD IL-2 between January 2005 to February 2012. Sixteen sites participated in the retrospective database. Patients were at least 18 years of age, had a diagnosis of mM or mRCC, and had received at least 1 dose of HD IL-2. Sites were encouraged to enroll consecutive eligible patients. In the current report, data from 362 patients, including 170 mM and 192 mRCC, were analyzed with the data extraction date of July 27, 2015. Available baseline data from enrolled patients included demographics (sex, age, and race) and clinical disease characteristics [Eastern Cooperative Oncology Group performance status (ECOG PS), disease stage, primary site of metastasis, *BRAF* mutation status in mM patients if tested, and prior treatment]. Patient eligibility at each site was confirmed by a study coordinator, and an electronic data capture system was used to record the data. The registry was approved by the Institutional Review Board at all participating sites. All data were subject to quality control procedures. Among retrospectively collected cohort, no consent was obtained, and only survival data were collected in follow-up. Additionally, due to the retrospective nature of data collection and exemption from consent, no information regarding subsequent IL-2 or other therapy post-IL-2 was collected.

### Treatment and assessments

Physicians managed and treated patients per each institution’s standard of care and their own clinical judgment. HD IL-2 (Proleukin^®^) was administered as an intravenous bolus every 8 h at a dose of 600,000 IU/kg or 720,000 IU/kg as tolerated, with up to 14 consecutive doses over 5 days (1 cycle of therapy). Patients received a second cycle of HD IL-2 after approximately a 9-day rest period, per the discretion of the investigator. Two cycles of HD IL-2 treatment constituted 1 standard course of HD IL-2 therapy. Additional courses were administered per the discretion of the treating physician. The duration of HD IL-2 drug administration was assessed from the time from the start of the first dose of HD IL-2 to the end of the last dose of HD IL-2 including rest periods. Response to HD IL-2 was determined by the investigator using either World Health Organization (WHO) criteria or Response Evaluation Criteria in Solid Tumors (RECIST), depending on the procedures utilized by the individual physician and site. Response was documented after each HD IL-2 treatment course (2 cycles) and approximately every 6 months upon conclusion of therapy

### Statistical analyses

All statistical analyses were performed using SAS software version 9.4. Patient characteristics, tumor response, and survival status were determined using data that were extracted on July 27, 2015. Frequency counts and measures of central tendency were performed to provide descriptive statistics. One-year, 2-year, and 3-year survival probabilities were obtained using the Kaplan–Meier product limit method and corresponding confidence interval (CI) were obtained using Greenwood’s formula. Kaplan–Meier curves with 95 % CIs were used to estimate mOS, with the log-rank test to determine significance (*P* < .05). OS was calculated from the date of first dose of HD IL-2 to date of death or date of most recent follow-up. Patients were followed until date of death or until last day of follow-up. Survival estimates for mRCC patients were also analyzed based on stratification into risk groups according to the International mRCC Database Consortium model (Heng criteria) [28]. The parameters included: ECOG PS ≥ 2, less than 1 year from initial diagnosis to treatment, hemoglobin concentration < lower limit of normal (LLN), calcium concentration > upper limit of normal (ULN), neutrophil count > ULN, and platelet > ULN. Patients were grouped into favorable (0 factors), intermediate (1–2 factors), and poor (3 or more factors) risk groups based on the number of risk factors.

## Results

### Patient population

Demographic and disease characteristics for both mM and mRCC patients are listed in Table 1. There were 91 % (*n* = 154) mM and 94 % (*n* = 89) mRCC patients that reported stage IV disease; note that 97 patients did not have tumor stage data available and were not used in this calculation. Most patients had an ECOG PS of 0 (*n* = 124, 73 % mM; *n* = 156, 82 % mRCC). Among the mM patients, only 57 (34 %) had *BRAF* mutation testing data available, and in this group, 40 tested positive for the *BRAF*V600E mutation. There were 58 (34 %) mM patients who had prior immunotherapy and 3 (2 %) who had prior targeted therapy. The database collected actual prior therapy, but did not specify whether prior immunotherapy was in the adjuvant setting. Among the mRCC patients, 183 (95 %) had a prior nephrectomy and 25 (13 %) had received prior targeted therapy. Thus, in this treatment time frame (2005–2012), HD IL-2 was often the initial treatment for eligible advanced mRCC and mM patients registered in PROCLAIM.

**Table 1:** Patient demographics and disease characteristics

	mM		mRCC		Total	
	*n* = 170	%	*n* = 192	%	*n* = 362	%
*Gender*						
Male	70	41	144	75	214	59
Female	100	59	48	25	148	41
*Age*						
<65	149	88	168	88	317	88
≥65	21	12	24	13	45	12
Median	54	NA	56	NA	55	NA
Range	20–79	NA	19–74	NA	19–79	NA
*Race*						
White	165	97	181	94	346	96
Black	4	2	2	1	6	2
Other	1	1	7	4	8	2
Decline	0	0	2	1	2	1
*ECOG PS* ^*a*^						
0	124	73	156	82	280	78
1	44	26	34	18	78	22
2	2	1	1	1	3	1
Missing^a^	0	0	1	1	1	0
*Stage* ^*b*^						
IIIc	8	5	NA	NA	8	3
III/III NOS	0	0	2	2	2	1
IIIb/IVa	6	4	4	4	10	4
IV/IVNOS	36	21	89	94	125	47
M1a	11	6	NA	NA	11	4
M1b	28	16	NA	NA	28	11
M1c	79	46	NA	NA	79	30
Other, specify	2	1	NA	NA	2	1
Missing^b^	0	0	97	NA	97	37

	mM		mRCC		Total	
*Had mutation testing*						
No	112	66	NA	NA	112	31
Yes^c^	58	34	NA	NA	58	16
*BRAF+*						
No^c^	17	30	NA	NA	17	30
Not tested^c^	1	2	NA	NA	1	2
Yes^c^	40	70	NA	NA	40	70
*Site of metastasis*						
Skin, lungs, LNs only	48	28	73	38	121	33
Other including skin, lungs, LNs	119	70	79	41	198	55
Not reported	3	2	40	21	43	12
*Prior therapy* ^*d*^						
Surgery	118	69	183	95	301	83
Radiation	49	29	15	8	64	18
Chemotherapy	32	19	5	3	37	10
Other immunotherapy	58	34	5	3	63	17
Targeted therapy	3	2	25	13	28	8
Blinded RCT	3	2	3	2	6	2
Other	3	2	1	1	4	1
Untreated	34	20	5	3	39	11

^a^ECOG PS based on patients with available data

^b^Sites were not required to enter in tumor stage for mRCC patients. Calculations were from patients with available data (95). Ninety-seven patients did not have tumor stage data

^c^BRAF mutation percentage was calculated from patients who had mutation testing

^d^Patients may have had multiple prior therapies. Percentages were calculated based on number of patients

*NA* not available, *ECOG PS* Eastern Cooperative Oncology Group performance status, *NOS* not otherwise specified, *LNs* lymph nodes, *RCT* research clinical trial

### IL-2 administration

The duration of HD IL-2 drug exposure was assessed from the start of the first dose of HD IL-2 to the end of the last dose of HD IL-2 including the rest period. For censored patients (patients still alive), the median duration of HD IL-2 drug exposure including the rest period was 2.4 months (range 0.56–14.37) and 0.66 months (range 0.06–12.99) for mM (*n* = 43) and mRCC (*n* = 89), respectively. Melanoma patients more frequently received additional courses (Supplementary Table 1). For all patients with mM (*n* = 170), the median duration of HD IL-2 drug exposure was 0.84 months (range 0.10–14.37), and for all patients with mRCC (*n* = 192), the median duration was 0.62 months (range 0.03–12.99). The number of doses of IL-2 per cycle for mM and mRCC are described in Supplementary Table 1 and were no different from current practice or recent clinical trials and clinical reports [5, 7].

### Tumor response

Response data were available for 158 patients (93 %) with mM: Eight patients (5 %) achieved CR, 16 (10 %) had PR, and 34 (22 %) had SD, while 100 (63 %) had progressive disease (PD) (Table 2). The objective response rate (ORR), defined as CR + PR, was 15 %, and the clinical benefit rate (CBR), defined as CR + PR + SD, was 37 %. For patients with mRCC, data were available for 185 (96 %) patients. There were 12 (6 %) patients with CR, 17 (9 %) with PR, 41 (22 %) with SD, and 115 (62 %) with PD (Table 2). The ORR in patients with mRCC was 15 %, and clinical benefit was observed in 70 (37 %) patients. CR, PR, or SD were frequently determined after course 2 of HD IL-2 treatment (≥62 % in mM and >80 % in mRCC), while 82 % of mM and 90 % of mRCC patients with PD were identified after course 1 of treatment (Table 2). Therefore, the efficacy of IL-2 can be determined in a relatively short period. Response rates were also analyzed based on prior treatment. Therapies prior to IL-2 are listed in Supplementary Table 2. Seventy-seven melanoma patients and 30 RCC patients received prior therapy, and this could have been in the adjuvant setting. No further details have been recorded. Some clearly received prior treatment for advanced disease. The majority of these patients received 1 or 2 prior therapies (Supplementary Tables 3, 4). In this database, there were no responses to IL-2 in patients who had 3 or more prior therapies.

**Table 2:** Summary and time to tumor response

	Final response	%	Course 1	Course 2	Course 3	Course 4	Course 5
mM *n* = (158)^a^							
CR	8	5	2	3	3	0	0
PR	16	10	8	4	4	0	0
SD	34	22	10	13	7	3	1
PD	100	63	82	12	6	0	0
CR + PR	24	15	10	7	7	0	0
CR + PR + SD	58	37	20	20	14	3	1
mRCC (*n* = 185)^a^							
CR	12	6	6	6	0	0	0
PR	17	9	3	11	2	0	1
SD	41	22	23	10	4	4	0
PD	115	62	103	8	4	0	0
CR + PR	29	15	9	17	2	0	1
CR + PR + SD	70	37	32	27	6	4	1

^a^Responses are from patients identified after each course of HD IL-2 therapy with a final response

### Survival data

**Fig. 1:**
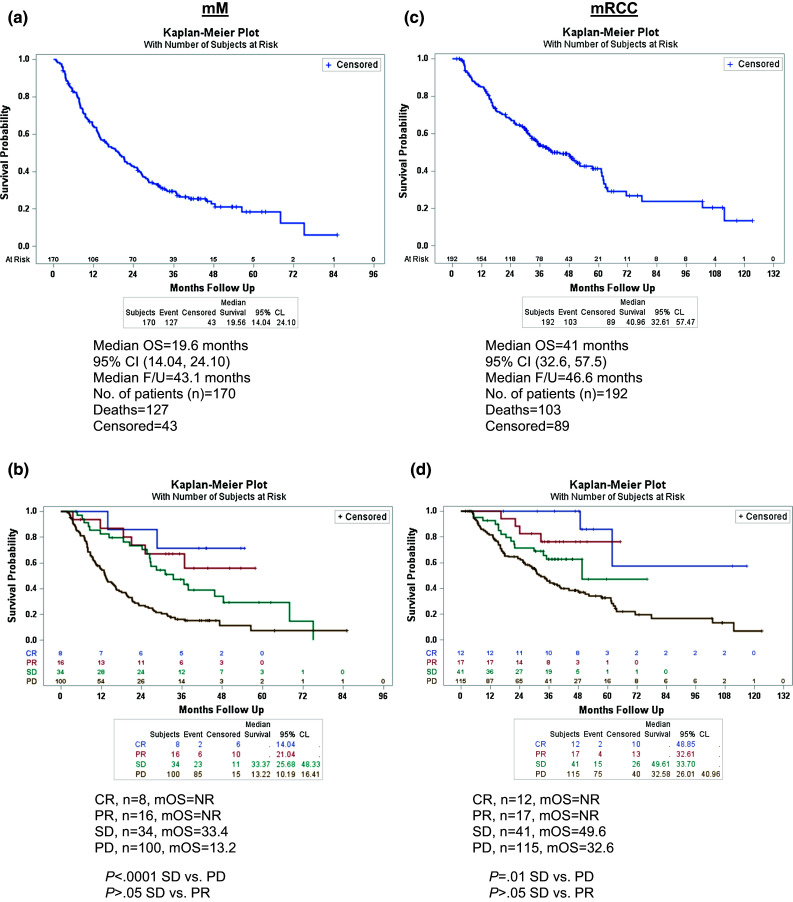
Overall survival in patients treated with HD IL-2 **a** Median OS (mM). **b** Median OS by response (mM). **c** Median OS (mRCC). **d** Median OS by response (mRCC). *Vertical bars* represent censored subjects. NR, not reached; F/U, follow-up

Survival estimates were calculated from the first dose of HD IL-2. For mM patients, the mOS was 19.6 months [95 % confidence interval (CI): 14.04, 24.10] with a median follow-up of 43.1 months (95 % CI 37.61, 46.13) (Fig. 1a). The mOS was not yet reached for those who experienced CR (*n* = 8) and PR (*n* = 16) (Fig. 1b). A statistically significant difference in mOS was observed between the patients who experienced SD and those who had PD (33.4 vs. 13.2 months; *P* < .0001) (Fig. 1b). There was no statistically significant difference in mOS between patients who experienced SD and PR. The 1-, 2-, and 3-year survival rates for patients with CR/PR were 91, 78, and 68 %, respectively. The 1-, 2-, and 3-year survival rates for patients with SD were 82, 73, and 47 %, respectively. An analysis of survival in patients who were systemic therapy-naïve versus those with prior systemic therapy was performed in the mM cohort. Prior systemic therapy included chemotherapy, targeted therapy, and/or immunotherapy. The mOS for patients without prior systemic therapy was 20.0 months (*n* = 93) compared to 18.7 months (*n* = 77) for those with prior systemic therapy (*P* = .76, Supplementary Figure 1a).

The mOS for patients with mRCC was 41 months (95 % CI 32.6, 57.5) with a median follow-up of 46.6 months (95 % CI 41.8, 49.6) (Fig. 1c). Patients with CR (*n* = 12) and PR (*n* = 17) had not reached a median overall survival, while patients with SD (*n* = 41) had a mOS of 49.6 months (Fig. 1d). There was no statistically significant difference in mOS between patients who experienced SD and PR. Patients with PD (*n* = 115) experienced a mOS of 32.6 months (Fig. 1d). There was a significant difference in mOS between the patients who experienced SD and those who had PD (49.6 vs. 32.6 months; *P* = .01 (Fig. 1d). The 1-, 2-, and 3-year survival rates for patients with CR/PR were 94, 84, and 77 %, respectively. The 1-, 2-, and 3-year survival rates for patients with SD were 93, 72, and 63 %, respectively. An analysis of survival in patients who were systemic therapy-naïve versus those with prior systemic therapy was performed in the mRCC cohort. The mOS for patients without prior systemic therapy was 48.9 months (*n* = 162) compared to 16.4 months (*n* = 30) for those with prior systemic therapy (*P* = .03, Supplementary Fig. 1b).

There were no reported investigator assessed treatment-related deaths for patients with mM or mRCC (*N* = 362).

### *BRAF* mutation status for mM patients

*BRAF* mutation testing was performed in only 57 of the total 170 melanoma patients. The mOS was 25.2 months for patients with *BRAFV600E* mutation (*n* = 40) and was 11.6 months for *BRAF* wild-type patients (*n* = 17) (data not shown). Of the patients with data available for response to IL-2, among those with *BRAF* mutation there were no CRs, 2 PR, 11 SD, and 25 PD, (data not shown). Among the *BRAF* wild-type patients, there were 3 CR, 2 PR, 1 SD, and 9 PD. However, because the proportion of patients tested is a small subset, this data are provided for completeness only.

### Survival by risk factors for mRCC

To determine OS based on externally validated prognostic risk factors for mRCC, patients were stratified into risk groups using the international mRCC Database Consortium model, also known as the Heng Criteria [28]. One hundred and forty-nine patients (78 %) had complete data for all six parameters and were included in this analysis. Thirty-seven patients (25 %) were in the favorable-risk group, 95 patients (64 %) were in the intermediate-risk group, and 17 patients (11 %) were in the poor-risk group. The mOS for patients in the favorable-risk group was 63.7 months, while for patients in the intermediate- and poor-risk groups, the mOS was 34.3 and 18.4 months, respectively (Table 3, *P* = .054 favorable vs. intermediate, *P* = .001 favorable vs. poor, *P* = 1.0 intermediate vs. poor). An analysis of response to HD IL-2 for the three risk groups was also performed (Table 3). For patients in the favorable group, there were 2 CR, 1 PR, 9 SD, and 25 PD. In the intermediate group, there were 9 CR, 4 PR, 17 SD and 60 PD. In the poor-risk group, there were 0 CR, 3 PR, 5 SD and 7 PD. Additionally, survival by risk category is presented in Table 3, with data from the Heng report [28] (targeted therapy) and the first Motzer report [29] (interferon therapy) provided for reference.

**Table 3:** Survival and response of mRCC to IL-2 (PROCLAIM) by Heng criteria

	Favorable (median survival, months)	Intermediate (median survival, months)	Poor (median survival, months)
PROCLAIM^a^	63.7 (*n* = 37)	34.3 (*n* = 95)	18.4 (*n* = 17)
Heng^b^	Not reached	27	8.8
Motzer^c^	20	10	4

PROCLAIM response by Heng model	Favorable		Intermediate		Poor	
	*N*	%	*N*	%	*N*	%
CR	2	5.4	9	9.5	0	0
PR	1	2.7	4	4.2	3	17.7
SD	9	24.3	17	17.9	5	29.4
PD	25	67.6	60	63.2	7	41.2
Missing	0	0	5	5.3	2	11.8
Total	37	100	95	100	17	100

Responses were based on patients with available data

^a^PROCLAIM median follow-up was 46.6 months

^b^Heng DY et al., JCO 2009, 27(34): 5794–9. Median follow-up was 24.5 months

^c^Motzer RJ et al., JCO 1999, 17(8): 2530–40. Median follow-up was 33 months

### Survival by time of HD IL-2 treatment

**Fig. 2:**
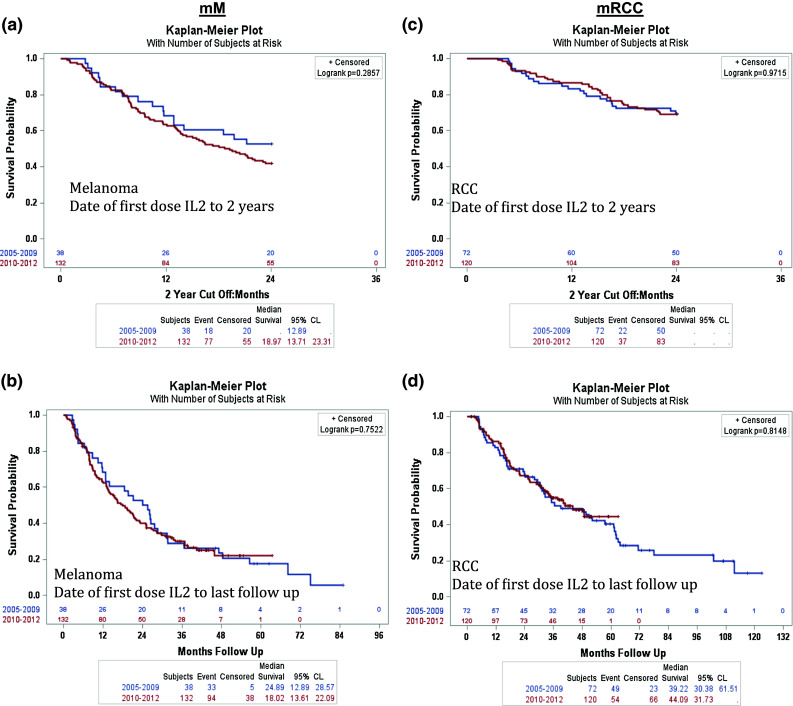
In order to determine the effect of targeted therapies on survival, patients were grouped into 2 cohorts based on years prior to or post targeted therapy approval (1 cohort from 2005–2009 and 1 cohort from 2010–2012). **a** Median OS for mM patients from date of first dose of IL-2 with a 2 year cutoff. **b** Median OS in mM patients from first dose of IL-2 to the last follow-up. **c** Median OS for mRCC patients from date of first dose of IL-2 with a 2 year cutoff. **d** Median OS in mRCC patients from first dose of IL-2 to the last follow-up

Although no data are available on post-IL-2 treatments, we have post hoc evaluated survival among cohorts of patients treated with HD IL-2 in the early years of the retrospective registry (2005–2009) and the later years (2010–2012), when more subsequent therapies were available. As can be seen in Fig. 2a, b, for mM and in Fig. 2c, d for mRCC, the survival is very similar.

## Discussion

The PROCLAIM observational database informs upon the real-world use and outcome of HD IL-2 for mM and mRCC patients in the era of TT and checkpoint immunotherapy. This retrospective analysis of contemporary patients with mM and mRCC receiving HD IL-2 revealed improved median overall survival compared to the historical experience. Single institution reports of IL-2 treated patients also show improvement in OS compared to historical experience [5, 30] as has a prospective multi-institutional study in mRCC (SELECT) [7]. Therefore, the PROCLAIM observational database demonstrates consistency with these reports [31, 32].

In this report, data were included from patients treated between 2005 and 2012, and even so, many of these patients received HD IL-2 as their initial treatment for advanced disease (mM no prior systemic treatment, *N* = 93; prior systemic treatment, *N* = 77; mRCC no prior systemic treatment, *N* = 162, prior systemic treatment, *N* = 30). However, they were treated during a transition period in which new agents were in development in clinical trials in both mRCC and mM, and eventually commercially, providing options for follow-on treatment. The clinical trials available to these patients in that era were either for patients who had progressed on prior treatment, or for newly diagnosed patients. Eventually, commercial drugs were available for patients with mRCC (2006) and for mM (2011).

As has been noted in clinical trials of the newer targeted therapies and more recently checkpoint inhibitors, survival of patients with advanced disease has improved, and patients are able to receive multiple sequential treatments with different mechanisms of action. Importantly, 43 % of HD IL-2-treated patients in this retrospective cohort achieved SD as best response. We have evaluated the outcome of treatment with HD IL-2 by best response and have observed prolonged OS in patients with SD, compared to patients with PD (Fig. 1b, d). We are exploring the potential impact of SD on OS as a consequence of HD IL-2 therapy in our prospective cohort versus simply the effect of follow-on therapy.

Because the data presented here reflect a retrospectively collected database, we do not have specific data on the treatment(s) patients received post-IL-2 progression. However, we have evaluated OS in the group treated with HD IL-2 between 2005 and 2009 versus between 2010 and 2012, possibly indicating that more subsequent therapy was available in the later time frame, but without information regarding the subsequent treatment of the patients in this retrospective cohort. As can be seen from Fig. 2a, b for melanoma and Fig. 2c, d for renal cell cancer, there is no significant difference in OS between the two time periods. This is surprising given the expected increased access to subsequent therapy in the later years. However, this information is derived from a retrospective database and could reflect selection bias of patients entered, small numbers in the earlier group, or possibly an effect of HD IL-2 on both groups. This will need to be the subject of future investigations, and no conclusions can be drawn from this dataset.

This retrospective cohort analysis provides a means of generating hypotheses to test in prospectively collected patient treatment records, which is ongoing. A major focus of the prospective PROCLAIM database is to evaluate the safety and efficacy of sequential treatments for mRCC and mM, and data on all treatment modalities are being collected, both prior and follow-on treatment. We will continue to evaluate the impact of SD on long-term outcome, as well as the potential for synergy among immunotherapy treatments. We will also evaluate the safety and efficacy of administration of HD IL-2 subsequent to the newer targeted and immunotherapy approaches.

Additional analyses of this retrospective cohort include an evaluation of the effect of prior therapy on outcome from IL-2. As noted in Supplementary Figure 1, there was a significant effect among patients with mRCC, but not significant among patients with mM. In part, this may be because much of the prior systemic treatment for mM was interferon and it was likely (not documented in the database) given as adjuvant therapy. However, this could also reflect a negative impact of prior therapy (mostly targeted therapy—*N* = 25 patients with prior targeted therapy) on outcome for patients with mRCC. Both of these hypotheses provide additional questions to pose from the PROCLAIM prospective cohort, and these analyses will be available with more detail from that cohort.

Although data on B-RAF mutation status were collected, it was not available for two-thirds of the subjects. Therefore, analysis of IL-2 outcome based on mutational status is only exploratory and will require prospective collection of this information. To date, there has been no evidence that B-RAF mutation status impacts the clinical response to HD IL-2 treatment.

Among the mRCC patients, prognostic subgroup information was evaluated, and in Table 3 we provide the response data to HD IL-2 by subgroup. Surprisingly, all subgroups have evidence of major response. Additionally, in Table 3, we have delineated the overall survival among the patients in this retrospective cohort by prognostic group alongside the results from the original reports by Heng et al. [28] of the factors among targeted therapy-treated patients and the original Motzer et al. [29] report in patients treated with interferon. It is gratifying to see that there is continued survival improvement over time with each successive report.

In summary, we report an analysis of a retrospectively accrued cohort from the PROCLAIM observational database of real-world patients treated in the modern era of HD IL-2 (2005–2012). We have demonstrated that these patients received similar dosing of HD IL-2 compared to clinical trials and that patient demographics reflect prior reports of these populations. Prior therapy may have an impact on outcome of mRCC patients subsequently treated with HD IL-2, compared with melanoma patients. Alternatively much of the prior therapy for the melanoma patients may have been in the adjuvant setting, such that prior treatment did not impact on effect of HD IL-2. Finally, patients achieving SD after IL-2 had improved OS compared to PD patients, as was also noted in the prospective IL-2 SELECT RCC trial [7]. We are evaluating the outcome of SD patients prospectively, and we are investigating the impact of sequential therapy and when these are given in the course of the disease (at SD or PD) among all IL-2 treated patients in the prospective cohort of the PROCLAIM observational database.

### Electronic supplementary material

Below is the link to the electronic supplementary material.

Supplementary material 1 (PDF 457 kb)

## Abbreviations

CBR: Clinical benefit rate
CI: Confidence interval
CR: Complete response
CTLA-4: Cytotoxic T lymphocyte antigen 4
ECOG PS: Eastern Cooperative Oncology Group performance status
HD IL-2: High-dose interleukin-2
LLN: Lower limit of normal
MAPK: Mitogen-activated protein kinase
mM: Metastatic melanoma
mOS: Median overall survival
mRCC: Metastatic renal cell carcinoma
mTOR: Mechanistic target of rapamycin
ORR: Objective response rate
PD: Progressive disease
PD-1: Programmed cell death receptor-1
PR: Partial response
RECIST: Response evaluation criteria in solid tumors
SD: Stable disease
TT: Targeted therapy
ULN: Upper limit of normal
VEGF: Vascular endothelial growth factor
WHO: World Health Organization
