# Supplementary material for: Contemporary experience with high-dose interleukin-2 therapy and impact on survival in patients with metastatic melanoma and metastatic renal cell carcinoma
Source: Cancer Immunol Immunother. 2016 Oct 6;65(12):1533–44. doi: 10.1007/s00262-016-1910-x (PMC5099373; doi:10.1007/s00262-016-1910-x)
Supplement: Supplementary file 1 — Supplementary material 1 (PDF 457 kb) [file 262_2016_1910_MOESM1_ESM.pdf]

Supplementary Table 1 Number of IL-2 doses

mM						mRCC					
Cycle #	N	Mean	Median	Minimum	Maximum	Cycle #	N	Mean	Median	Minimum	Maximum
1	170	9.37	9	3	14	1	191	8.78	9	1	15
2	157	7.83	8	1	13	2	174	6.64	6	1	14
3	60	8.7	9	2	14	3	52	8.35	8	2	14
4	56	7	7	1	14	4	48	5.96	6	1	11
5	24	8.92	9	4	14	5	15	7.73	7	5	14
6	24	5.75	5.5	1	14	6	13	5.23	5	3	8
7	5	7.6	8	4	9	7	5	5.6	5	4	7
8	3	8	8	7	9	8	5	5.2	5	4	7
9	2	9	9	8	10	9	1	5	5	5	5
10	2	8.5	8.5	7	10						
11	1	10	10	10	10						
12	1	9	9	9	9						

Supplementary Table 2 Therapies prior to IL-2

Prior Therapy	mM (N)	mRCC (N)
Only Chemotherapy	19	3
Only Immunotherapy	44	1
Only Targeted Therapy	0	21
Chemotherapy & Immunotherapy	11	1
Chemotherapy & Targeted Therapy	0	1
Immunotherapy & Targeted Therapy	1	3
Chemotherapy & Imuunotherapy & Targeted Therapy	2	0
Total	77	30

Supplementary Table 3 Response to IL-2 by prior therapy for mM

# of Prior Therapy	CR (N)	PR (N)	SD (N)	PD (N)	Missing (N)	Total (N)
1	3	3	10	38	1	55
2	0	2	6	9	1	18
3 or more	0	0	1	3	0	4

Prior systemic treatments include chemotherapy, immunotherapy (including IFN-a), targeted therapy. It does not include radiation, surgery, and other treatments.

Supplementary Table 4 Response to IL-2 by prior therapy for mRCC

# of Prior Therapy	CR (N)	PR (N)	SD (N)	PD (N)	Missing (N)	Total (N)
1	1	1	3	13	1	19
2	0	0	2	5	2	9
3 or more	0	0	0	2	0	2

Prior systemic treatments include chemotherapy, immunotherapy (including IFN-a), targeted therapy. It does not include radiation, surgery, and other treatments.

Supplementary Figure 1

a

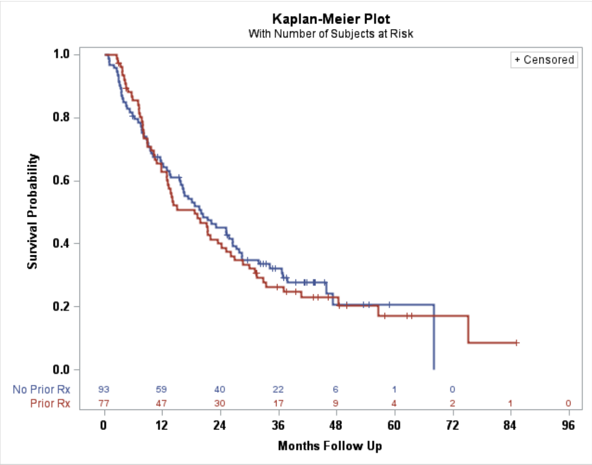

b

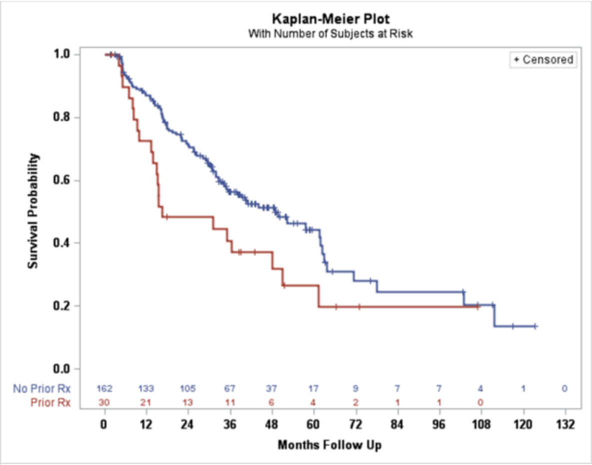

	n	mOS	95%CI	
No Prior RX	93	20	15.45,26.5	
Prior RX	77	18.67	12.89, 24.1	
p-value=.7642				

	n	mOS	95%CI	
No Prior RX	162	48.85	34.26, 61.84	
Prior RX	30	16.44	13.41, 48.0	
p-value=.0335				

Supplementary Figure 1 a Overall survival in mM patients with and without prior systemic therapy. **b** Overall survival in mRCC patients with and without prior systemic therapy. Prior systemic therapy included chemotherapy, targeted therapy, and/or immunotherapy.
